# The Amending Bill

**Published:** 1913-07-12

**Authors:** 


### NATIONAL INSURANCE ACT DEPARTMENT.

### LX.—The Amending Bill (*continued*).

It is proposed in the Amending Bill to the National Insurance Act, see Article LIX., to repeal the section which provides for reduced benefits for persons entering insurance within one year of the commencement of the Act, who were aged fifty years and upwards, by substituting the same level benefit (10s. a week for men and 7s. 6d. a week for women) as for younger persons. Space did not allow us to add that the other benefits of the Act beside sickness benefit are also to be given to these old people. Employed persons above sixty-five years of age when the Act came into operation are not included under its provisions as ordinary insured persons. Their benefits are at present such as the approved societies care to offer. No reserves are set up, and instead of the Government contributing as a proportion of the benefits paid, it is adding 2d. to each weekly contribution. While, for these reasons, the approved societies have had nominally the right to give whatever benefits they like to persons over sixty-five years of age, they have mostly adopted one or other of the alternative scales issued as suggestions by the Insurance Commissioners. The first schemes put forward showed very small amounts of sickness benefit, and did not provide for medical benefit beyond the age of seventy. In fact, under the first scheme no medical benefit of any description was given, and this has *been considered a real hardship by most of the old people.*

### Medical Benefit for Persons over Sixty-five.

Under the provisions of the Amending Bill, when it becomes law, persons above sixty-five years of age at entry into insurance will not only be insured for the full sickness and disablement benefits which will in any case cease at the age of seventy, but they will also have the right to medical treatment and attendance for the rest of life. There is only one small exception to this rule, and that is that at least fifty weekly contributions must have been paid by or in respect of such a person. This is a limitation that is desirable in its intention, and is, of course, designed to preclude the provision of medical benefit for the rest of life, costing approximately 10s. a year, when perhaps only a few pence had actually been paid in contributions. In actual practice the limitation is likely to cause irritation, and saving in cost will hardly be worth the trouble and expense it will entail. There must, of course, be some limit. The limit imposed by the Act is the attainment of the age of seventy, and we suggest that it is in harmony with the proposals of the Amending Bill to extend to all persons who have had to pay contributions under the Act and who are in employment at the age of seventy the right to medical treatment and attendance for the rest of life. To be really effective, this clause should be made retrospective so far as medical benefit is concerned, so that persons who have already passed out of insurance by the attainment of the age of seventy may share the advantages now to be conferred on those who are still contributing.

### Employer's Portion of Arrears to be Excused.

Objection has been raised in certain quarters against the compulsory nature of the National Insurance Act, and it has been suggested that its benefits should be open to those who voluntarily care to avail themselves of them, and that compulsion be eliminated. Those who have any experience of the industrial classes will realise the necessity for clear and explicit regulations when dealing with industrial organisations, and that the voluntary system cannot attain to national proportions. The failure of the voluntary system is exemplified in regard to the question of arrears of contributions as laid down in the Act. At the present time an approved society is empowered to excuse the payment of any part of the arrears, which may have accrued, due by or in respect of a member during unemployment, provided such part does not exceed the amount which would have been paid on his account by his employer. It is to be clearly noted that the Act allows such payment to be excused if the approved society should see fit to do so; but no compulsion is placed on any society to excuse arrears or any part thereof. In the circumstances it is hardly to be wondered at that, as the societies are looking to the future and realising the necessity for preserving every possible source of income, they have resolutely declined to excuse the payment of the employer's share of contributions in arrear. It is quite clear that while any important society declined to give such a concession no other important society could do so on purely competitive grounds as a business enterprise. This would, doubtless, have been foreseen if there had been exhaustive discussion of the section in Parliament.

The failure of the approved societies to relax their requirement for the payment of arrears in full has led to the inclusion in the Amending Bill of a proposal by virtue of which when a member falls into arrear through unemployment he will only be required to make good his own contributions, and will not have to pay the sum which would otherwise have been paid by his employer.

### The Advantage to Insured Persons.

In industrial insurance of every description the necessity for the regular payment of contributions and the paying up of arrears when these are allowed to accrue admittedly entails hardship in many cases. This hardship is particularly evident when the insurance is compulsory and when, in addition to his own contributions which are in arrear, the insured persons has also to make good the payments which, if he had been so fortunate as to be in employment, would have been paid by his employer. Thus the proposal of the Amending Bill in regard to excusing the payment of the employer's contribution when arrears have accrued will give substantial relief to the insured person and should be welcomed accordingly.

Arrears of contributions accruing during the first year after the commencement of the Act were specially exempted, and do not therefore count in the calculation of the average arrears which are subsequently to form the basis of the reduction of benefits. In point of fact it would not be possible for benefits to be reduced on account of arrears until October next, that is, until the fifth quarter has been completed. Thus the provision as to arrears in the present Amending Bill is opportune, and the apparent hardship of the Act as now in force will not have time to take effect.

### Effect on Finances of Approved Societies.

The effect of the new proposal will, of course, be to reduce to a certain extent the income of the approved societies. It has to be remembered, however, that the original actuarial estimates were made to allow for an average of three weekly contributions a year in arrear on account of each insured person, and therefore that if the arrears do not exceed this average the societies will not actually suffer loss on the basis of those estimates. The proposal will in consequence merely entail the sacrifice of a profit to the societies which would have been gained at the expense of their unemployed members. So far as can be judged on the basis of the cards already sent in there is little prospect of the arrears amounting on the average to anything like three weeks a year. If, however, they should by any chance exceed that limit, provision is made for the society, or branch, on the production of evidence of such excess to be recouped out of the fund retained by the Insurance Commissioners on account of the initial reserves. In the event of the sum required exceeding £100,000 a year the excess is to be provided by Parliament.

### Benefit for Exempted Persons.

By Clause 4 it is proposed that medical and sanatorium benefits shall be provided for those persons who are exempted from insurance, but on account of whom their employers have to pay the weekly contribution of 3d. No benefits were provided for these persons under the National Insurance Act, but the Insurance Commissioners were empowered to make regulations as to the disposal of the fund derived from their contributions for their benefit in the event of them becoming employed contributors. The powers of the Insurance Commissioners in this connection were extremely limited. There was no power to enable them to draw on Parliamentary funds, and in consequence the benefits that could be given would necessarily have had to be limited in value to the amount of the employers' contributions. At the present time we are informed, in the Chief Actuary's report, there are in England about 94,000 exempted persons. It is, however, anticipated that this number will be reduced to about 75,000, and for the whole of Great Britain it is estimated that there will be 90,000 exempted persons. On this basis the cost to the State, over and above the employer's contributions, to enable medical and sanatorium benefits to be given, will be £19,000 a year. In view of the fact that exempted persons have to show to the satisfaction of the Insurance Commissioners that they are provided for in event of sickness without recourse to the benefits of the Act, it is an anomaly that so large a sum of money should be required from Parliament to furnish benefits which, *ex hypotheosi*, are not required.

The payment of contributions in respect of exempted persons by their employers was inserted in the Act to prevent employers from endeavouring to obtain exempted persons as employees instead of insured persons. Experience of the working of the Act clearly shows that so small an advantage as 3d. a week would have little or no influence on employers of labour when engaging their workpeople. It seems to us, therefore, that it would be preferable to amend the Act by repealing the section under which contributions have to be paid in respect of exempted persons rather than by the suggested provision of benefits which will cost the nation £19,000 a year on the basis of the Chief Actuary's estimate.

### Modifications of Sickness Benefit.

There are two proposals in the Bill for altering the conditions under which sickness benefit may be claimed. At present, if there are two periods of sickness which are not separated by twelve months during which at least fifty weekly contributions have been paid, the second period will rank as a continuation of the first period, and sickness benefit will cease at the termination of a total period of twenty-six payments. It is now proposed that there shall be the one requirement only, namely, that at least twelve months must elapse between two periods of attack, otherwise they will rank as the same illness, and the requirement as to the payment of fifty contributions is dispensed with.

The other proposal is that when the insured person is entitled to compensation on account of accident or industrial disease and in consequence only receives a part of the sickness benefit payment, the period as for which the payment of benefit is to rank, in calculating the term for which it is to continue, is to be proportional to the ratio borne by the amount of benefit paid to that which would otherwise have been payable.

These are apparently slight modifications, but they are important in that they remove grounds for possible dissatisfaction at comparatively slight cost, and, further, that they will considerably ease the administration of the benefit.

### Answers to Correspondents.

"M.B." informs us that he and his assistant at a hospital are not on the local panel, but are asked every day to sign about a dozen certificates to enable patients to obtain sickness benefit from their approved societies. He asks if he should demand a nominal fee for so doing.—Panel doctors are required to furnish certificates of this description free of cost by the terms of their agreements. If "M.B." and his assistant wish to spare themselves the trouble of signing certificates they should charge a fee. The insured persons will then go to their panel doctors.

